# Formulation, Characterization, and Preliminary In Vitro Evaluation of Letrozole Incorporated Eudragit^®^ RS 100 Nanoparticles Using HepG2 Cells

**DOI:** 10.2174/0113816128474280260720051151

**Published:** 2026

**Authors:** Muhammet Ali Polat, Kadir Aykaç, Zerrin Cantürk, Ebru Başaran

**Affiliations:** 1 Anadolu University, Institute of Graduate Education, 26470, Eskisehir, Türkiye;; 2 Department of Pharmaceutical Technology, Faculty of Pharmacy, Anadolu University, 26470, Eskisehir, Türkiye;; 3 Department of Pharmaceutical Technology, Faculty of Pharmacy, Erzincan Binali Yıldırım University, Erzincan, Türkiye;; 4 Department of Pharmaceutical Microbiology, Faculty of Pharmacy, Anadolu University, 26470, Eskisehir, Türkiye

**Keywords:** Letrozole, hepatocellular carcinoma, Eudragit^®^ RS 100, polymeric nanoparticles, HepG2 cells, encapsulation efficiency

## Abstract

**Introduction/objective:**

Letrozole (LTZ)-loaded polymeric nanoparticles (PNPs) were formulated with Eudragit^®^ RS100 to investigate their potential as a preliminary drug delivery system for hepatocellular carcinoma (HCC).

**Methods:**

Nanoparticles were prepared using the spray-drying technique with a Büchi B-90 Nano Spray Dryer and characterized in terms of morphology, particle size, polydispersity index (PDI), zeta potential, drug loading efficiency, thermal and structural characteristics, *in vitro* release behavior, and preliminary cytotoxicity.

**Results:**

Scanning electron microscopy (SEM) analysis demonstrated the formation of predominantly spherical particles with relatively smooth surfaces. The prepared nanoparticles exhibited initial particle sizes ranging between 253 nm and 425 nm, with PDI values of 0.3–0.4 and positive zeta potential values between 36 and 48 mV. Encapsulation efficiency (EE%) and drug loading (DL%) values were determined as 47.9%–56.9% and 9.1%–18.6%, respectively. Thermal and structural analyses indicated the molecular dispersion of LTZ within the polymeric matrix. *In vitro* release studies conducted at pH 7.4 demonstrated an initial burst release, followed by a sustained drug release profile over 24 h, with cumulative drug release reaching almost 80%. The preliminary cytotoxicity of the formulations was evaluated using the methylthiazolyl-diphenyl-tetrazolium bromide (MTT) assay on human hepatocellular carcinoma (HepG2) and healthy human dermal fibroblast (BJ) cell lines. Free LTZ demonstrated an IC_50_ value of 142.76 μg/mL against HepG2 cells, whereas the optimized nanoparticle formulation exhibited an IC_50_ value higher than 121.85 μg/mL.

**Discussion:**

The obtained results confirmed the successful development of LTZ-loaded polymeric nanoparticles with suitable physicochemical properties, efficient drug encapsulation, and sustained release behavior. The positive surface charge and nanoscale size may support formulation stability. Furthermore, the nanoparticles preserved the cytotoxic activity of LTZ, indicating their potential as an effective drug delivery system.

**Conclusion:**

Overall, the findings suggest that the developed nanoparticles constitute a promising drug delivery system for further investigation in HCC-related therapy. However, additional mechanistic studies and *in vivo* evaluations are required to comprehensively assess their true therapeutic potential.

## 1 INTRODUCTION

Cancer remains one of the leading causes of death worldwide and a major challenge to public health as the number of new cancer cases and cancer-related deaths increases. It is estimated that approximately 10 million people die from cancer annually [1]. Conventional therapeutic approaches, including chemotherapy, radiotherapy, surgery, and immunotherapy, form the basis of current cancer treatment strategies [2]. Although these treatments provide significant benefits, they also come with numerous drawbacks, such as serious side effects, relapse, drug-resistant cancers, and a lack of therapeutic success [3]. Consequently, cancer research remains prominent in both biological and medical research, and the quest for better diagnostic and treatment options for cancer patients continues [4].

Nanoparticles provide a great strategy to enhance the delivery of therapeutic agents due to their nanoscale size, which enables safe intravenous administration, efficient tissue penetration, and passive tumor targeting *via* the enhanced permeability and retention (EPR) effect, while also facilitating interactions with cellular targets [5-8]. Therefore, nanoparticles have emerged as a key platform in the development of advanced and targeted drug delivery systems for cancer therapy owing to their ability to protect therapeutic agents and enable site-specific delivery. Their advantages are clearly demonstrated in controlled drug delivery applications in many medical disciplines, including but not limited to oncology, ophthalmology, cardiology, endocrinology, immunology, and gene therapy [6-8]. Polymeric nanoparticles (PNPs) have become highly recognized as effective carriers for the prolonged and controlled release of anticancer drugs in nanocarrier systems. Therapeutic agents in polymer-based nanomedicine can be either chemically bonded to or physically embedded within the PNP matrix, with pH-responsive coatings, ligand-decorated nanoparticle surfaces, and multilayered and stimuli-sensitive release mechanisms enabling enhanced interactions with target sites [9-11]. PNPs improve the solubility, bioavailability, and systemic circulation time of therapeutic agents while exhibiting low toxicity, minimal immunogenicity, and negligible inflammatory responses, thus representing safe and effective drug delivery vehicles [12].

Letrozole (LTZ) is a third-generation, non-steroidal aromatase inhibitor approved by the FDA for postmenopausal breast cancer treatment [13]. Anti-estrogen agents for breast cancer management are commonly prescribed, and LTZ is among the most common anti-estrogen agents prescribed for breast cancer treatment. The role of estrogen in the development of hepatocellular carcinoma (HCC) remains controversial, as it regulates liver metabolism and energy homeostasis *via* estrogen receptors and influences liver morphology and function, potentially contributing to the development of various liver diseases, including cancer. While LTZ has proven to be clinically effective mostly in estrogen-related cancers, recent studies suggest that aromatase inhibitors may exert anticancer effects through alternative pathways, including modulation of oxidative stress and inhibition of proliferative signaling cascades, which may be relevant in non-classical targets such as HCC. However, LTZ has also been associated with various adverse effects, such as therapeutic resistance and treatment failure [14, 15]. Due to these limitations, several advanced drug delivery systems, namely liposomes, hydrogels, nanoparticles, and nanoniosomes, have been developed to modulate LTZ release, reduce systemic adverse effects, and enhance therapeutic efficacy [16, 17].

This study aimed to formulate PNPs to enhance the therapeutic efficacy of LTZ while minimizing the adverse effects by using lower LTZ doses. Characterizations of the nanoparticles were conducted to determine the morphological characteristics, particle size, size distribution (polydispersity index; PDI), zeta potential, and *in vitro* release profile. Structural analyses of the nanoparticles were conducted using differential scanning calorimetry (DSC), Fourier transform infrared spectroscopy (FT-IR), and proton nuclear magnetic resonance (^1H-NMR) analyses.

The present study was designed as a formulation-development and preliminary *in vitro* screening study rather than as a definitive therapeutic efficacy investigation. The primary objective was to formulate and characterize LTZ-loaded Eudragit^®^ RS 100 polymeric nanoparticles produced by the nano spray drying method and to evaluate their physicochemical properties, short-term stability, and preliminary cytotoxicity against HepG2 cells. Because the role of estrogen signaling and aromatase inhibition in HCC remains complex and not fully established, LTZ was used here primarily as a hydrophobic model anticancer compound with a clinically known pharmacological profile. The biological findings are therefore interpreted cautiously and are intended to guide future mechanistic and liver-specific validation studies.

## 2 MATERIALS AND METHODS

### 2.1 Materials

In this study, LTZ was obtained from Sigma (Germany). Eudragit^®^ RS 100 was purchased from Evonik Industries (Germany). Acetone, acetonitrile, deuterochloroform, ethanol, calcium chloride dihydrate, methanol, potassium chloride, and sodium bicarbonate were obtained from Merck (Germany). All other chemicals used were of analytical grade.

### 2.2 Formulation of Nanoparticles

The spray drying method was selected for the preparation of PNPs. Process parameters, including feed rate, aspiration rate, nozzle diameter, and inlet temperature, were optimized [18-20]. A constant amount of Eudragit^®^ RS 100 was dissolved in a solvent mixture (acetone and methanol; 36:4 *v/v*), and LTZ was added to the solution at different concentrations relative to the polymer weight. After achieving homogeneous and transparent solutions, spray drying was performed using a Büchi B-90 Nano Spray Dryer (Switzerland) with an inlet temperature of 105°C ± 5°C (outlet temperature of 52°C ± 3°C). Placebo nanoparticles were formulated without the addition of LTZ. The collected particles were stored in well-closed containers until analysis.

### 2.3 Characterization Studies of the Nanoparticles

#### 2.3.1 SEM Analyses

The surface characteristics and morphology of the nanoparticles were analyzed using scanning electron microscopy (SEM) with a Carl Zeiss Ultra Plus (Germany).

#### 2.3.2 Particle Size, Size Distribution, and Zeta Potential Analyses

Prior to particle size and zeta potential measurements, approximately 5 mg of each dried nanoparticle formulation was dispersed in distilled water at a final concentration of approximately 1:100 (*w/v*). The dispersion was vortexed for 60 s and allowed to equilibrate at room temperature. To minimize the influence of visible aggregates on dynamic light scattering measurements, the dispersion was passed once through a 0.45 μm polytetrafluoroethylene (PTFE) filter mounted on a 5 mL disposable syringe. The first drops were discarded, and the filtrate was immediately transferred into disposable measurement cuvettes for particle size, PDI, and zeta potential analyses. Measurements were performed using a Malvern Zetasizer Nano ZS (UK). All analyses were performed in triplicate (with an average of 20 runs per analysis). Thermal analyses were performed using a Shimadzu DSC 60 (Japan).

#### 2.3.3 Thermal Analyses

For thermal evaluations, accurately weighed samples were placed in aluminum cells, which were then firmly closed. Analyses were performed over the range of 30°C to 300°C with a heating rate of 10°C/min. An empty aluminum cell was used as a reference. DSC thermograms of pure LTZ and pure polymer were used as references for the evaluation of structural changes during the formulation stages.

#### 2.3.4 Fourier Transform Infrared Spectroscopy Analyses

FT-IR analyses of the formulations were conducted over 4000–400 cm⁻¹ using a Perkin-Elmer Spectrum 2000 (UK). FT-IR spectra of pure LTZ and pure polymers were used as references for evaluating structural changes during the formulation stages.

#### 2.3.5 Nuclear Magnetic Resonance Spectroscopy Analyses

Ultra Shield CP MAS NMR (Brucker, Germany) was used for ¹H-NMR spectroscopy analysis. Both FT-IR and ¹H-NMR analyses were performed at 25°C ± 2°C. ¹H-NMR analysis spectra of pure LTZ and pure polymers were used as references for the evaluation of structural changes during formulation stages.

### 2.4 Quantitative Analyses

Drug loading (DL%) and encapsulation efficiency (EE%) were determined to evaluate the amount of drug incorporated into the nanoparticles, using a modified and validated high-performance liquid chromatography (HPLC) method [21]. Briefly, for the determination of the total LTZ amount (DL%), 5 mg of each nanoparticle formulation was dissolved in 5 mL of methanol, and the solutions were analyzed by HPLC following proper dilutions. DL% of the nanoparticles was calculated according to Equation 1. For EE% analyses, 5 mg of each nanoparticle formulation was dispersed in 5 mL of isopropyl alcohol and vortexed for 1 min for complete dissolution of LTZ adsorbed on the particle surface. The amount of encapsulated LTZ was calculated by subtracting the surface-associated drug from the total LTZ content according to Equation 2. Analyses were performed in triplicate.

DL% = (mass of LTZ quantified in nanoparticles / total mass of nanoparticles analyzed) × 100 (Eq. 1)

EE% = [(mass of LTZ quantified in nanoparticles- mass of LTZ quantified on the surface of nanoparticles)] / mass of LTZ quantified in nanoparticles) × 100 (Eq. 2)

### 2.5 In Vitro Release Studies and Release Kinetics

The release profiles of LTZ from the nanoparticles were assessed in a phosphate-buffered solution (pH 7.4) at physiological temperature (37°C ± 2°C) with constant stirring at 150 rpm using a dialysis membrane with a 14 kDa molecular weight cut-off (Sigma, Germany). The samples were withdrawn at specific time intervals over 24 hours and analyzed *via* HPLC to construct the release profiles. All experiments were performed in triplicate. The *in vitro* release data were evaluated using the DDSolver software program to identify the best-fitting kinetic model describing the release behavior of LTZ from Eudragit^®^ RS 100 nanoparticles [22].

### 2.6 In vitro Cytotoxicity and Anticancer Activity Studies

*In vitro* cytotoxic activity of the formulations was evaluated by the methylthiazolyl-diphenyl-tetrazolium bromide (MTT) assay. Briefly, 1 × 10^4^ cells per well were seeded in 96-well plates and incubated overnight. After treatment with various sample concentrations for 24 hours, 20 µL of MTT solution (5 mg/mL in PBS) was added to each well and incubated for 4 hours at 37°C ± 1°C. The formed formazan crystals were dissolved in dimethyl sulfoxide (DMSO), and absorbance was measured at 570 nm using a microplate reader. Cell viability (%) was calculated relative to the untreated control, and IC_50_ values were determined through one-way ANOVA analysis using the GraphPad Prism program. For the MTT analysis, human healthy BJ cells and human HCC (HepG2) cell lines were obtained from the American Type Culture Collection (ATCC). Cells were cultured in Dulbecco's Modified Eagle Medium (DMEM) supplemented with 10% Fetal Bovine Serum (FBS) and 1% penicillin–streptomycin at 37°C in a humidified 5% CO_2_ atmosphere. Each sample (prepared PNP formulations, Eudragit^®^ RS 100, and LTZ) was dissolved in DMSO and then diluted with culture medium to final concentrations ranging from 15.6 µg/mL to 500 µg/mL. The final DMSO concentration did not exceed 0.5% (*v/v*). All experiments were conducted in three independent replicates, with eight samples analyzed in each replicate (n = 8). Data were presented as the mean ± standard deviation (SD). Statistical analysis was conducted using one-way ANOVA followed by Tukey’s post hoc test, with *p* < 0.05 considered statistically significant. Since only commercially available cell lines were used, no ethical approval was required for the study.

### 2.7 Stability Studies

Stability studies of the prepared nanoparticles were conducted by storing the samples in stability chambers at 5°C ± 3°C, 25°C ± 2°C (with 60% ± 5% RH), and 40°C ± 2°C (with 75% ± 5% RH) in accordance with ICH Q1A guidelines [23]. Stability assessments were carried out at predetermined time intervals (initial, 1^st^ month, and 3^rd^ month) with the analyses of particle size, PDI, zeta potential, and the DL%. One-way ANOVA analysis with the Graphpad Prism program was used for statistical evaluations

## 3 RESULTS AND DISCUSSION

### 3.1 Formulation of Nanoparticles

In the present study, the Büchi-B90 Nano Spray Dryer was used to produce nanoparticles. Prior to production, critical process parameters, including polymer concentration, feed rate, aspiration rate, inlet temperature, and nozzle diameter, were optimized to ensure desirable physicochemical characteristics [19]. The concentration of Eudragit^®^ RS 100 was kept constant while LTZ was incorporated into the polymer solutions at different concentrations (10%, 15%, and 20% LTZ relative to the polymer weight), considering the significant impact of the drug-to-polymer ratio on the DL% and EE% of the nanoparticles (Table **1**) [20]. LTZ and Eudragit^®^ RS 100 were dissolved in a mixture of acetone and methanol (36:4 *v/v*). The inlet temperature was maintained at 105°C ± 5°C, while the outlet temperature was 52°C ± 3°C. The resulting dry particles were collected directly using an electrostatic particle collector. Conventional spray dryers such as the Büchi-190 require a relatively large amount of solution, and the process yield often remains below 70%, representing a significant limitation, especially for high-value drugs. However, the Büchi-B90 Nano Spray Dryer overcomes most of the major drawbacks of conventional spray dryers through its vibrating nozzle, laminar heating, and electrostatic particle collection, achieving yields of up to 90% [18]. Although the theoretical yield of the method was very high, as expected, the actual yield of the collected particles was relatively low, most likely due to electrostatic repulsive forces between charged particles during the nanoparticle collection stage. Collected dry particles were stored in well-closed containers until analysis. Placebo nanoparticles were prepared without the addition of LTZ and used as reference formulations for the evaluation of structural changes during the spray drying process. The compositions of the formulations and critical process parameters are presented in Table **1**.

### 3.2 Characterization Studies of the Nanoparticles

#### 3.2.1 SEM Analyses

SEM analysis was performed to evaluate the surface morphology and structural characteristics of the prepared nanoparticles. SEM is considered a robust and suitable technique for the characterization of polymeric nanoparticles, particularly for the evaluation of particle morphology, surface topology, and aggregation behavior [24]. SEM images revealed that placebo nanoparticles exhibited hollow and partially collapsed structures with wrinkled surfaces, commonly associated with rapid solvent evaporation during the spray drying process (Fig. **1**). In contrast, LTZ-loaded nanoparticles displayed comparatively more spherical morphology with smoother surfaces, suggesting that the incorporation of LTZ influenced particle formation and surface characteristics. In addition, no visible LTZ crystals were detected on the surface of the nanoparticles, indicating successful encapsulation of the drug within the polymeric matrix (Fig. **1**) [25]. Although the presence of some larger particles and aggregates was observed, a substantial population of submicron-sized nanoparticles was clearly detected throughout the formulations (Fig. **1**).

#### 3.2.2 Particle Size, Size Distribution, and Zeta Potential Analyses

SEM analysis revealed the presence of larger particles and agglomerates inherent to spray-dried systems (Fig. **1**). Therefore, prior to particle size and PDI analyses, samples were dispersed in distilled water at an approximate concentration of 1:100 (*w/v*), and the dispersions were filtered through a 0.45 µm PTFE membrane filter prior to measurement in order to minimize the presence of large aggregates and ensure reliable particle size determination using the dynamic light scattering method, which is highly sensitive to oversized particles and aggregates. In this study, submicron particles, preferably below 400 nm, with PDI values indicating an acceptable but not necessarily monodisperse distribution, were considered suitable for preliminary formulation screening. Although particle sizes in the range of approximately 100–200 nm are often considered favorable for passive tumor accumulation, the present formulations should not be interpreted as optimized for *in vivo* tumor targeting. Rather, the obtained size range supports their use as polymeric nanoparticulate carriers for further *in vitro* evaluation [26, 27].

Particle size analysis confirmed that aggregates and larger particles were effectively removed by filtration through a 0.45 µm membrane filter (Table **2**). Particle sizes ranged from 253 nm to 314 nm, indicating that LTZ incorporation influenced particle size, most likely through the formation of more spherical structures, as confirmed by particle size analysis (Fig. **1**, Table **2**).

The zeta potential values ranged from +36.1 mV to +48.4 mV on the day of production, indicating the colloidal stability of the particles (Table **2**). Cationic nanoparticles interact electrostatically with negatively charged cancer cell membranes, promoting enhanced cellular uptake *via* endocytosis and internalization relative to neutral or anionic nanoparticles. Studies have revealed that cationic nanoparticles are considered highly effective in both targeting and treating HCC in preclinical studies. They are frequently used as delivery vehicles for chemotherapy, siRNA, and gene therapy, providing improved drug stability and tumor accumulation [28, 29].

#### 3.2.3 Thermal Analyses

Phase transitions during the formulation stages were evaluated using DSC to investigate potential drug–polymer interactions and the molecular dispersion within the polymeric matrix [30, 31]. The melting peak of pure LTZ was detected at 185°C, revealing the typical crystalline nature of the drug in accordance with former studies [30]. Pure Eudragit^®^ RS 100 and the placebo formulation were in an amorphous state with no significant endothermic peak appearance. The presence of the characteristic melting peak of LTZ was observed in the thermogram of the physical mixture (ERS100+LTZ), indicating the absence of significant interaction between the drug and the polymer. The disappearance of the characteristic melting peak of LTZ in the ML1, ML2, and ML3 thermograms suggested that the drug was molecularly dispersed within the polymer matrix, leading to the formation of an amorphous structure (Fig. **2**) [30-34].

#### 3.2.4 Fourier Transform Infrared Spectroscopy Analyses

Characteristic peaks for pure LTZ included N-C amine (1138 cm⁻¹), aliphatic C-H (1436.04 cm⁻¹), aromatic C=C (1600–1475 cm⁻¹), C=N (1690–1640 cm⁻¹), weak aromatic C-H (1728.22 cm⁻¹), nitrile C≡N (2231.64 cm⁻¹), amine salt N-H (2951.09 cm⁻¹), and moderate aliphatic C-H (3118.90 cm⁻¹) were observed [35, 36]. In accordance with the literature, Eudragit^®^ RS 100 showed characteristic peaks at 1147.65 cm⁻¹ (C–O), 1436.97 cm⁻¹ (CH_3_), and 1728.22 cm⁻¹ (C=O) (Fig. **3**) [37]. The characteristic peaks of pure LTZ were not detected in the formulation spectra, indicating that the drug was molecularly dispersed in the polymer matrix without any new signal formation, suggesting no chemical interactions among the formulation components, consistent with DSC analysis (Figs. **2** and **3**) [38, 39].

#### 3.2.5 Nuclear Magnetic Resonance Spectroscopy Analyses

Molecular structure, chemical integrity, drug–polymer interactions, and the incorporation of active compounds into the polymeric matrix were assessed by ^1H-NMR analysis [40]. The ^1H-NMR spectra (500 MHz, CDCl_3_) of pure Eudragit^®^ RS 100 and the ML-PL formulation exhibited nearly identical signals, indicating the structural integrity of the polymer during the formulation process (Fig. **4**) [40]. The characteristic signals in the 7–8 ppm region were assigned to the aromatic protons of LTZ, detected at δ ppm: 8.09 (s, 1H), 8.06 (s, 1H), 7.70 (m, 4H), 7.28 (m, 4H), and 6.81 (s, 1H) [41]. These signals were also revealed in the spectra of the drug-loaded formulations (highlighted with arrows), confirming the absence of chemical interactions between LTZ and the polymer (Fig. **4**).

### 3.3 Quantification of LTZ

Quantitative analysis of LTZ-loaded nanoparticles was performed using a modified HPLC method [21]. The DL% of the nanoparticles was found to be 9.66%, 14.37%, and 18.62% for ML1, ML2, and ML3, respectively. To determine the EE% of the nanoparticles, the surface-located LTZ amounts were analyzed, and, according to Equation 2, % EE values were calculated. Analysis results showed that EE% values were 56.9 ± 3.8, 47.9 ± 0.4, and 49.2 ± 1.1 for ML1, ML2, and ML3, respectively. Nearly 50% of the total LTZ was located on the surface of the particles (Table **2**).

### 3.4 In Vitro Release Study

The *in vitro* release study was performed at 37°C ± 2°C in pH 7.4 phosphate buffer using a dialysis membrane at a stirring speed of 150 rpm to evaluate the release behavior of LTZ from Eudragit^®^ RS 100 nanoparticles as an initial screening condition [41-44]. Considering the acidic microenvironment associated with tumor tissues, the physiological medium does not fully reproduce the complexity of systemic circulation or the HCC tumor microenvironment [43, 44]. However, Eudragit^®^ RS100 is a water-insoluble, pH-independent polymer with a controlled swelling rate; therefore, the release profiles were primarily governed by its swelling behavior and diffusion-controlled mechanisms within the polymer matrix [45].

The analysis results revealed that, depending on the amount of drug located on the particle surface (Table **2**), an initial burst release occurred within the first 2 h, reaching approximately 30% cumulative release (Fig. **5**). The release profile of pure LTZ was used as a reference, showing approximately 93% release after 24 h. In comparison, ML1, ML2, and ML3 formulations exhibited cumulative release rates of 76.8%, 73.9%, and 76.9%, respectively, at the end of 24 h. These results indicate that LTZ-incorporated nanoparticles provided sustained release over 24 h, which can be attributed to the pH-independent swelling behavior of Eudragit^®^ RS100 (Fig. **5**) [44, 46].

### 3.5 Evaluation of Drug Release Kinetic Models

The evaluation of drug release kinetics and mechanisms from pharmaceutical nanoparticles plays a crucial role in the characterization of nanoparticulate drug delivery systems. The application of kinetic models is particularly valuable in elucidating the mechanisms that govern drug release, which can be used to control release profiles [47]. Various mathematical models have been employed to assess the drug release behavior from delivery systems. These models serve as essential tools in the development of new formulations, providing insight into drug release processes under both *in vitro* and *in vivo* conditions [48, 49]. In this study, the DDSolver software was used to analyze the release kinetics of the drug from nanoparticles. The results of the kinetic modeling analysis are presented in Table **3**. Based on the obtained data, models with the lowest Akaike Information Criterion (AIC) and highest Model Selection Criterion (MSC), along with the highest coefficient of determination (r²) and release rate constant (K) values, were selected as the most suitable release models [50]. The release profile of LTZ was best described by the first-order kinetic model, indicating a release mechanism primarily governed by the solubility of the pure drug (Table **3**). The Korsmeyer-Peppas model was the best fit for all formulations (Table **3**). The Korsmeyer-Peppas model is a simple model specifically designed to describe the release of a drug molecule from a polymeric matrix through a combination of diffusion and erosion/swelling of the polymeric matrix, in accordance with the swelling properties of Eudragit^®^ RS 100 at physiological pH [44, 46].

The Korsmeyer-Peppas model is also an empirical model that can explain release systems according to Fick's law [51, 52]. By evaluating the "n" values obtained in Table **3**, the diffusion behavior of the active ingredient released from the nanoparticles according to Fick's law was investigated. According to Fick's law, systems that exhibit appropriate diffusion should have "n" values less than 0.45 [53]. In our study, the "n" values obtained for all formulations were found to be less than 0.45, thus confirming that the formulations exhibited appropriate diffusion according to Fick's law (Table **3**) [54].

### 3.6 In vitro Cytotoxicity and Anticancer Activity Studies

In the present study, LTZ was used as a model hydrophobic compound to evaluate the nanoparticle delivery system's performance and to conduct preliminary assessments of its potential as a therapeutic agent for HCC. Previous studies with nanoparticle-based systems loaded with antitumor agents have shown that pathways such as reactive oxygen species generation, mitochondrial integrity disruption, caspase activation, and inhibition of signaling pathways involved in cell proliferation (MAPK/ERK and mTOR) may contribute to cytotoxic responses in HepG2 cells [55–57].

LTZ is known to inhibit aromatase (CYP19A1), leading to decreased estrogen biosynthesis [58]. This estrogen deprivation is a key mechanism underlying the suppression of estrogen-dependent tumor growth through inhibition of estrogen-mediated proliferative signaling. However, due to the context-dependent and complex role of estrogen in cancer biology—where it is often associated with tumor progression, particularly in breast cancer—the effect of estrogen modulation on HepG2 cells remains controversial. The mechanisms by which estrogen reduction may induce cytotoxicity in HepG2 cells, rather than promoting proliferation as observed in other malignancies, are still not fully elucidated [59, 60].

Among the formulations evaluated, ML3 exhibited significantly higher cytotoxicity against HepG2 cells compared to ML1 and ML2 (*p* < 0.05), while maintaining minimal toxicity toward BJ fibroblast cells (IC_50_ > 500 µg/mL) (Table **4**). These findings indicate a degree of selective cytotoxicity, which is particularly important in anticancer research, especially for liver-related applications where minimizing damage to normal cells is essential. The lower IC_50_ value of ML3 compared to free LTZ suggests improved efficacy of the nanoparticle formulation under *in vitro* conditions (Table **4**).

The observed selective cytotoxicity of ML3 compared to BJ cells may indicate a favorable safety profile; however, since the current study is limited to *in vitro* cytotoxicity analyses, further studies are required for detailed molecular mechanisms responsible for the observed effects on the normal liver cell lines (such as THLE-2 or primary hepatocytes) for better evaluation of the tissue-relevant selectivity and to strengthen the translational relevance of the analyses results.

### 3.7 Stability Studies of the Nanoparticles

The nanoparticles were placed in tightly closed containers and stored under three different conditions: 5°C ± 3°C; 25°C ± 2°C (60% ± 5% RH); and 40°C ± 2°C (75% ± 5% RH). Short-term stability studies were performed by evaluating changes in particle size, PDI, zeta potential, and LTZ content [24]. According to the results, the samples stored at 25°C ± 2°C (60% ± 5% RH) and 40°C ± 2°C (75% ± 5% RH) exhibited slightly greater variations in particle size and PDI values than those stored at 5°C ± 3°C, as expected. However, these differences were not statistically significant (*p* > 0.05) (Table **5**). The largest particle size was observed for the ML3 formulation stored at 40°C ± 2°C (75% ± 5% RH), reaching 425 nm after 1 month of storage. However, the particle size of the same formulation decreased to 372 nm at the end of the 3rd month, suggesting that the initial increase can most likely be attributed to aggregated nanoparticles in the analyzed sample. Particle size analyses were performed as quickly as possible due to the pH-independent swelling properties of Eudragit^®^ RS100 [44, 46].

The zeta potential values of the particles remained above +30 mV throughout the 3-month storage period, with no significant changes (*p >* 0.05), indicating the colloidal stability of the particles. LTZ incorporation influenced the cationic characteristics of the nanoparticles; however, even the formulations most susceptible to degradation maintained high positive zeta potential values, ranging from +38.4 ± 1.5 to +46.7 ± 1.6 mV after 3 months of storage at 40 ± 2°C (75 ± 5% RH). These findings suggest that the formulations retained their cationic surface properties and colloidal stability throughout the storage period (Table **5**).

For the ML1, ML2, and ML3 formulations stored at 5°C ± 3°C, the initial LTZ contents were 9.66%, 14.37%, and 18.62%, respectively. After 3 months of storage, these values remained stable at 9.51%, 14.42%, and 18.73%, respectively. Similarly, nanoparticles stored at 25 ± 2°C (60 ± 5% RH) exhibited LTZ contents of 9.62%, 14.43%, and 18.71%, whereas those stored at 40 ± 2°C (75 ± 5% RH) showed LTZ contents of 9.58%, 14.46%, and 18.71% at the end of the 3-month storage period. No statistically significant changes in LTZ content were observed under any storage condition (*p >* 0.05), indicating that the incorporated LTZ remained stable throughout the 3-month study period (Table **5**).

Although no marked loss of LTZ content was observed during the 3-month storage period, the accelerated stability condition revealed formulation-related physical changes, particularly for the ML3 formulation. The increase in particle size from 314 nm at day 0 to 425 nm after 1 month at 40 ± 2°C (75 ± 5% RH), together with the increase in PDI, suggests partial particle aggregation. Although the particle size decreased to 372 nm at the end of the 3rd month, refrigerated or controlled room-temperature storage appears to be more appropriate at this preliminary stage, while accelerated conditions may be more useful for guiding further formulation optimization.

### 3.8 Limitations and Future Perspectives

The present study has several limitations that should be considered when interpreting the findings. First, the biological evaluation was limited to MTT-based viability screening, and no mechanistic assays were performed to determine whether the observed HepG2 response was associated with apoptosis, cell-cycle arrest, oxidative stress, mitochondrial dysfunction, aromatase inhibition, or other molecular pathways. Second, BJ fibroblasts were used as a general non-cancerous control; however, normal liver-relevant cell models, such as THLE-2 cells or primary hepatocytes, are required to evaluate hepatic selectivity more appropriately. Third, the *in vitro* release study was limited to 24 h and was conducted only in pH 7.4 phosphate buffer, which does not fully reflect serum-containing systemic conditions or the tumor microenvironment. Fourth, the physical changes observed under accelerated storage conditions, especially for ML3, indicate that further formulation optimization may be necessary to improve long-term colloidal stability. Finally, no pharmacokinetic, biodistribution, tumor penetration, or *in vivo* toxicity data were generated. Therefore, the present work should be regarded as a formulation-development and preliminary *in vitro* screening study that provides a basis for future mechanistic, liver-specific, and *in vivo* investigations.

## CONCLUSION

In this study, LTZ-loaded Eudragit^®^ RS 100 PNPs were successfully prepared using the nano spray-drying method and characterized in terms of morphology, particle size, PDI, zeta potential, drug loading, thermal and structural characteristics, molecular interactions, *in vitro* release characteristics, preliminary *in vitro* cytotoxicity, and short-term stability for 3 months. Among the formulations prepared, ML3 showed the highest LTZ loading and the strongest cytotoxic response against HepG2 cells while maintaining low toxicity toward BJ fibroblasts under the tested conditions. These findings suggest that Eudragit^®^ RS 100 nanoparticles may serve as a useful carrier platform for LTZ and justify further investigation in HCC-related experimental models. However, the present results do not demonstrate therapeutic efficacy and should not be interpreted as evidence of clinical applicability. Future studies should include mechanistic assays, liver-relevant normal cell controls, extended and biorelevant release testing, improved accelerated stability assessment, and *in vivo* pharmacokinetic and safety evaluations.

## Figures and Tables

**Fig. (1):**
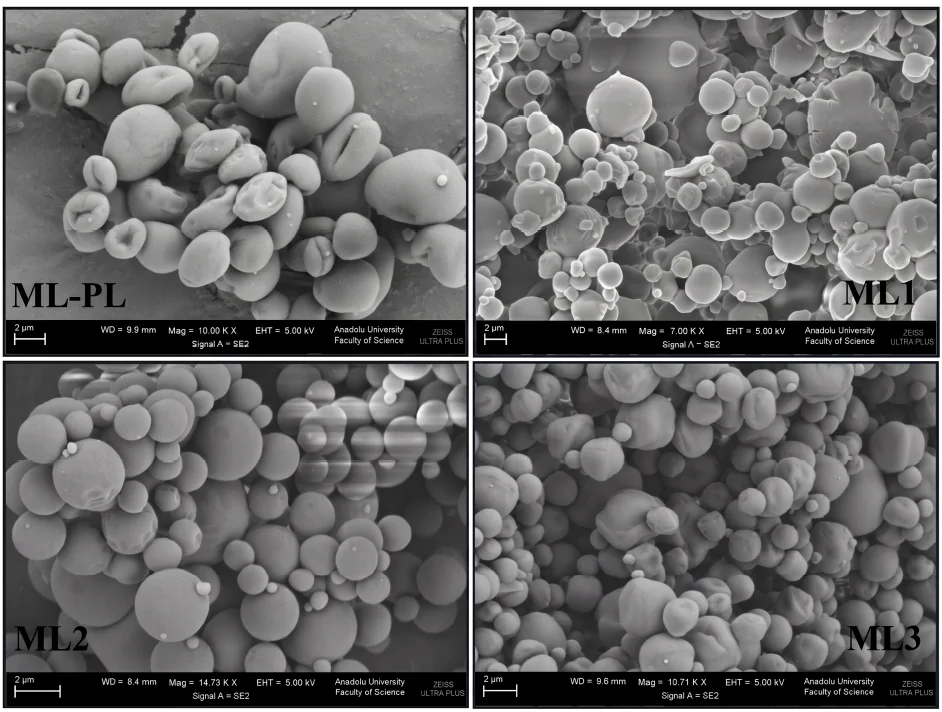
SEM microimages of the nanoparticles prepared.

**Fig. (2):**
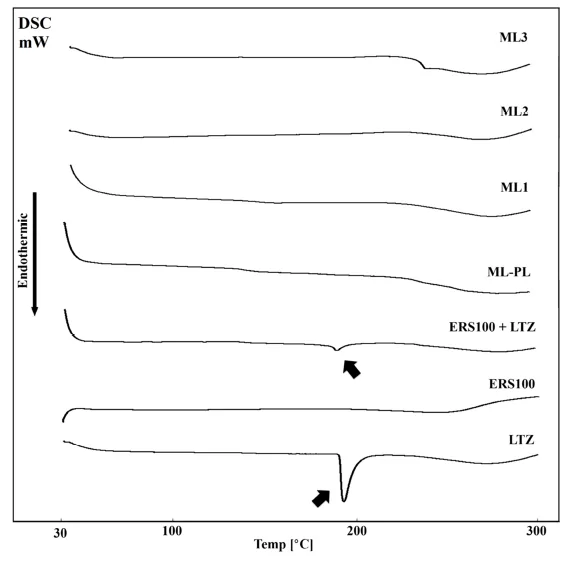
Thermograms of pure materials, physical mixture (ERS100+LTZ) and the nanoparticles prepared [ERS100: Eudragit^®^ RS 100].

**Fig. (3):**
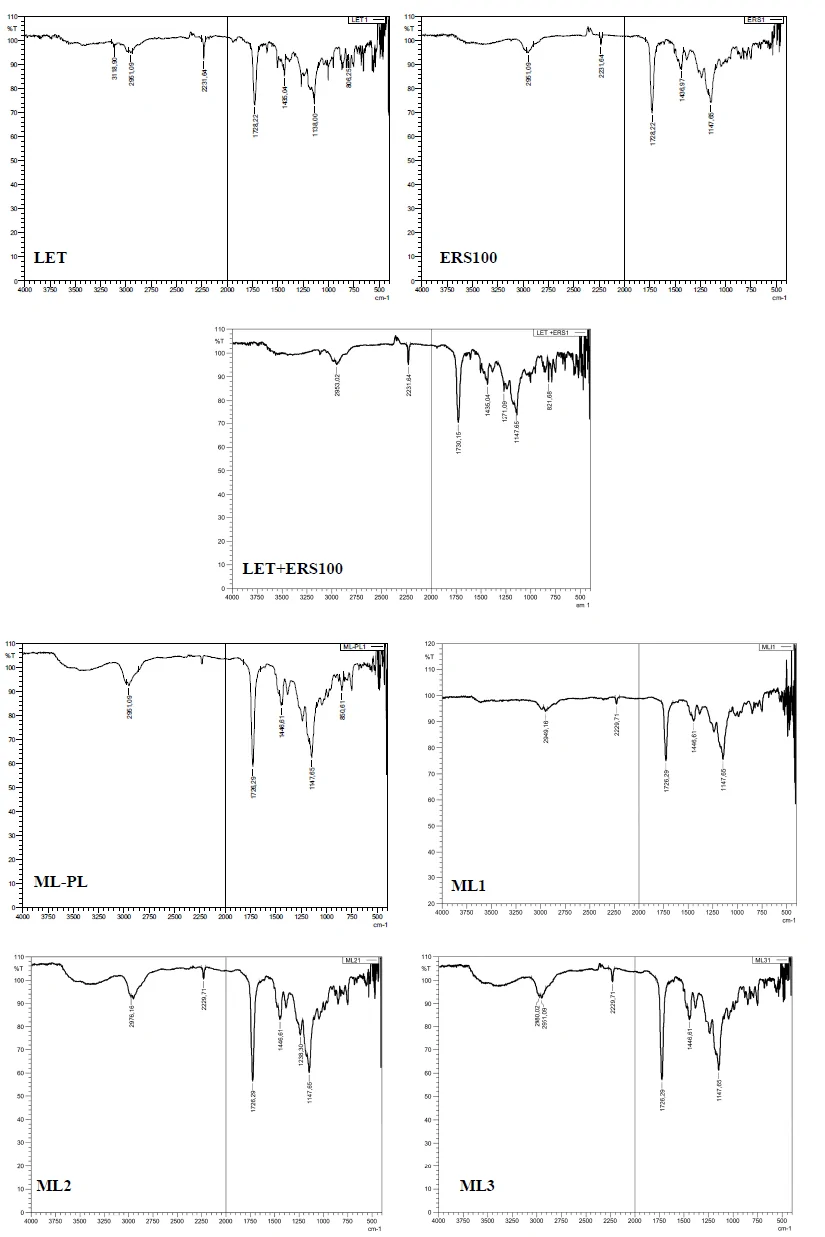
FT-IR spectra of pure materials, physical mixture (ERS100+LTZ) and the nanoparticles prepared [ERS100: Eudragit^®^ RS 100.

**Fig. (4):**
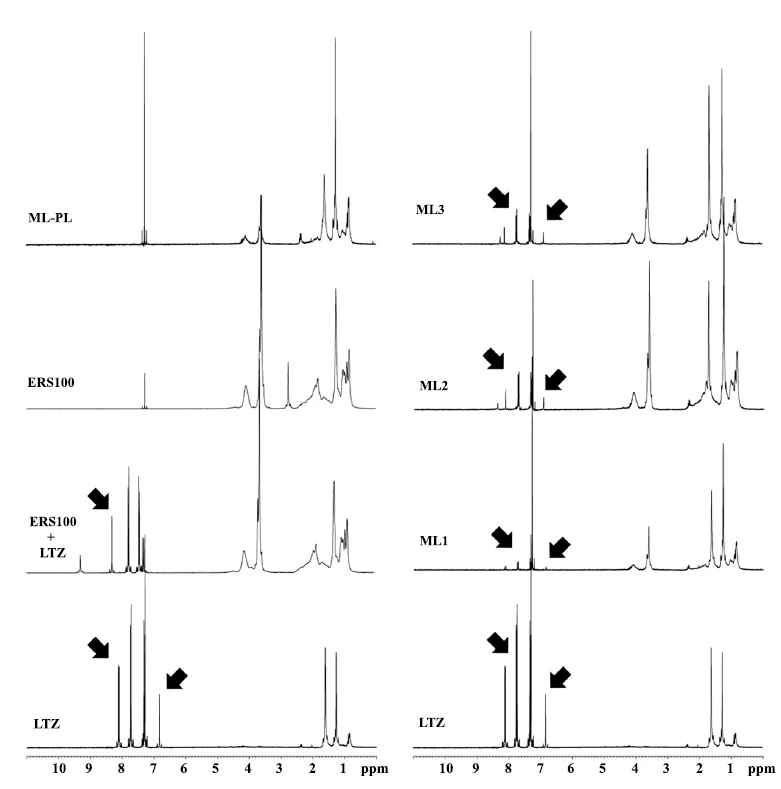
1H-NMR spectra of pure materials, physical mixture (ERS100+LTZ) and the nanoparticles prepared [ERS100: Eudragit^®^ RS 100.

**Fig. (5):**
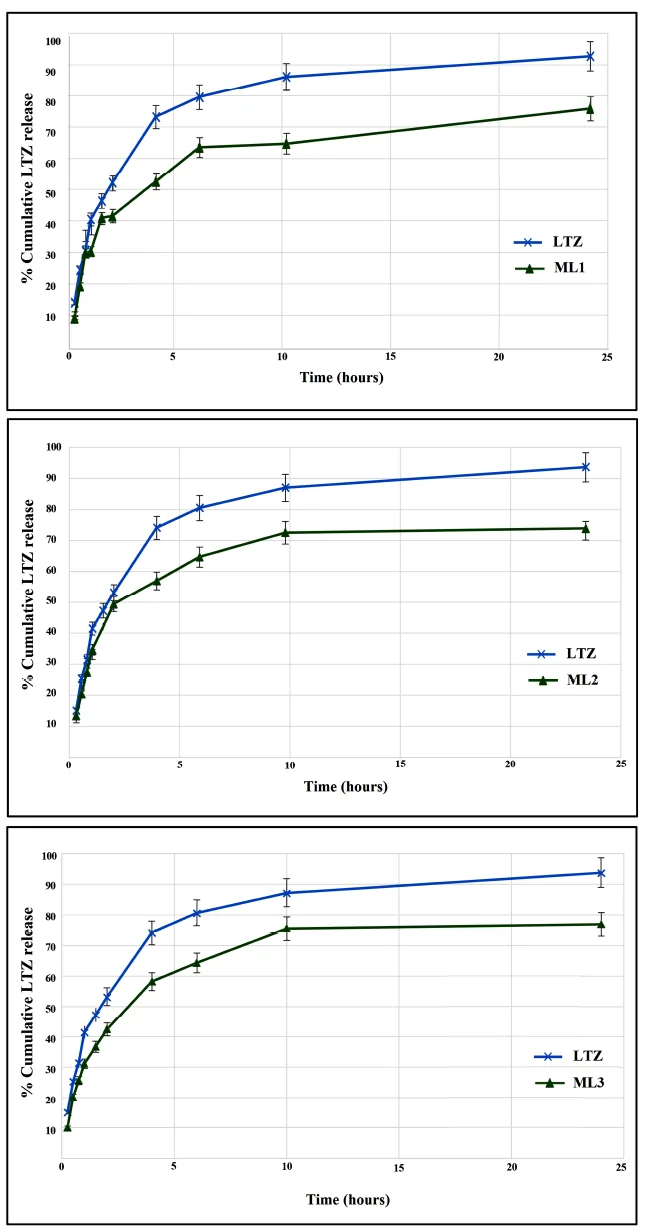
*In vitro* release analysis results of the pure LTZ *versus* nanoparticles prepared (Mean ±SE; n=3).

**Table 1:** Compositions of the formulations and process parameters.

**Code**	**Eudragit^®^RS 100 (g)**	**LTZ (mg)**	**Drug-to-polymer Ratio (%; *w/w*)**
**ML-PL**	2	-	-
**ML1**	2	200	10
**ML2**	2	300	15
**ML3**	2	400	20
**Process Parameters for Büchi B-90 Nano Spray Dryer**			
**Inlet temperature (°C)**	105 ± 5		
**Outlet temperature (°C)**	52 ± 3		
**Feed rate/pump rate (%)**	20–40		
**Drying gas flow rate (mL/min)**	100-120		
**Spray mesh size (µm)**	7.0		
**Aspirator rate (%)**	100		
**Solvent system (*v/v*)**	Methanol: acetone; 4:36		
**Drying gas type**	Air		

**Table 2:** Particle size, PDI, zeta potential, and LTZ content analysis results of the nanoparticles prepared (n=3, mean ± SD).

**Code**	**Particle Size (nm)**	**PDI**	**Zeta Potential (mV)**	**DL% (% *w/w*)**	**EE% (% *w/w*)**
**ML-PL**	253 ± 20.4	0.30 ± 0.04	48.4 ± 1.3	-	-
**ML1**	279 ± 10.2	0.30 ± 0.07	47.2 ± 0.6	9.11 ± 0.03	56.9 ± 3,8
**ML2**	280 ± 15.0	0.40 ± 0.03	47.2 ± 0.2	14.37 ± 0.02	47.9 ± 0.4
**ML3**	314 ± 7.5	0.44 ± 0.03	36.1 ± 1.5	18.62 ± 0.05	49.2 ± 1.1

**Table 3:** Analysis of *in vitro* release kinetics model fitting for the prepared nanoparticles.

**Code**	**Criteria**	**Zero-Order**	**First Order**	**Higuchi**	**Hixson-Crowell**	**Korsmeyer-Peppas**	**Hopfenberg**
**ML1**	**K**	4.501	0.216	20.728	0.051	**32.577**	0.000
	**r^2^**	-1.0027	0.5432	0.5917	0.3624	**0.9033**	0.4860
	**AIC**	92.1783	77.3975	76.2767	80.7331	**62.6978**	79.4005
	**MSC**	-0.8945	0.5836	0.6957	0.2500	**2.0536**	0.3833
	**n**	-	-	-	-	**0.298**	-
**ML2**	**K**	4.542	0.251	21.275	0.054	**34.696**	0.000
	**r^2^**	-1.3107	0.5587	0.4684	0.3231	**0.8663**	0.5031
	**AIC**	93.7637	77.2087	79.0698	81.4860	**66.0916**	79.2158
	**MSC**	-1.0375	0.6180	0.4318	0.1902	**1.7297**	0.4172
	**n**	-	-	-	-	**0.281**	-
**ML3**	**K**	4.649	0.229	21.337	0.053	**32.180**	0.000
	**r^2^**	-0.7104	0.7506	0.6686	0.6071	**0.8881**	0.7194
	**AIC**	92.4824	73.2279	76.0699	77.7729	**66.0381**	75.2299
	**MSC**	-0.7368	1.1887	0.9045	0.7342	**1.9077**	0.9885
	**n**	-	-	-	-	**0.317**	-
**LTZ**	**K**	5.643	**0.402**	26.119	0.063	40.906	0.000
	**r^2^**	-0.9759	**0.9270**	0.5886	0.5846	0.8853	0.9178
	**AIC**	97.0826	**64.1019**	81.389	81.4878	69.4375	66.1115
	**MSC**	-0.8810	**2.4170**	0.6883	0.6784	1.8835	2.2161
	**n**	-	-	-	-	0.300	-

**Table 4:** IC50 values of ML1, ML2, ML3, Eudragit^®^ RS 100 and positive control LTZ in HepG2 and BJ cell lines.

**Code**	**BJ Cell Line IC_50 _(µg/mL)**	**HepG2 Cell Line IC_50 _(µg/mL)**
**Control**	>500	>500
**ML1**	>500	495.03 ± 0.04
**ML2**	>500	384.39 ± 0.08
**ML3**	>500	121.85 ± 1.02
**Eudragit^®^ RS 100**	>500	>500
**LTZ**	>500	142.76 ± 0.65

**Table 5:** Particle size, PDI, zeta potential, and total LTZ content of formulations during the storage period (Mean ± SD, n = 3).

**Time**	**5°C ± 3°C**					**25°C ± 2°C (60% ± 5% RH)**				**40°C ± 2°C (75% ± 5% RH)**			
	**Code**	**Particle Size (nm)**	**PDI**	**Zeta Potential (mV)**	**Total LTZ Content(% *w/w*)**	**Particle Size (nm)**	**PDI**	**Zeta Potential (mV)**	**Total LTZ Content (% *w/w*)**	**Particle Size (nm)**	**PDI**	**Zeta Potential (mV)**	**Total LTZ Content (% *w/w*)**
**Day 0**	**ML-PL**	253 ± 20.4	0.30 ± 0.04	+48.4 ± 1.3	-	253 ± 20	0.30 ± 0.04	+48.4 ± 1.3	-	253 ± 20	0.30 ± 0.04	+48.4 ± 1.3	**-**
	**ML1**	279 ± 10.2	0.30 ± 0.07	+47.2 ± 0.6	9.66 ± 0.03	279 ± 10	0.30 ± 0.07	+47.2 ± 0.6	9.66 ± 0.03	279 ± 10	0.30 ± 0.07	+47.2 ± 0.6	9.66 ± 0.03
	**ML2**	280 ± 15	0.40 ± 0.03	+47.2 ± 0.2	14.37 ± 0.02	280 ± 15	0.40 ± 0.03	+47.2 ± 0.2	14.37 ± 0.02	280 ± 15	0.40 ± 0.03	+47.2 ± 0.2	14.37 ± 0.02
	**ML3**	314 ± 7.5	0.44 ± 0.03	+36.1 ± 1.5	18.62 ± 0.05	314 ± 7.5	0.44 ± 0.03	+36.1 ± 1.5	18.62 ± 0.05	314 ± 7.5	0.44 ± 0.03	+36.1 ± 1.5	18.62 ± 0.05
**1^st^ Month**	**ML-PL**	260 ± 12.6	0.40 ± 0.2	+47.2 ± 0.7	-	245 ± 15.2	0.34 ± 0.03	+44.3 ± 1.1	-	220 ± 17.4	0.37 ± 0.03	+49.4 ± 0.5	-
	**ML1**	290 ± 5.2	0.30 ± 0.06	+46.5 ± 0.6	9.50 ± 0.04	285 ± 10.5	0.38 ± 0.01	+43.7 ± 1.4	9.53 ± 0.03	288 ± 13.8	0.42 ± 0.05	+45.8 ± 1.7	9.55 ± 0.02
	**ML2**	305 ± 7.4	0.50 ± 0.04	+44.7 ± 1.4	14.45 ± 0.03	297 ± 14.6	0.53 ± 0.02	+42.5 ± 0.9	14.35 ± 0.04	315 ± 16.7	0.62 ± 0.02	+46.2 ± 1.6	14.32 ± 0.06
	**ML3**	370 ± 16.4	0.30 ± 0.05	+42.4 ± 0.8	18.69 ± 0.03	347 ± 19.7	0.60 ± 0.04	+39.6 ± 1.3	18.71 ± 0.02	425 ± 11.6	0.54 ± 0.03	+38.7 ± 1.2	18.58 ± 0.04
**3^rd^ Month**	**ML-PL**	270 ± 17.8	0.45 ± 0.05	+46.4 ± 1.2	-	275 ± 20	0.38 ± 0.06	+48.8 ± 1.2	-	276 ± 5.7	0.41 ± 0.03	+47.8 ± 0.8	-
	**ML1**	290 ± 18.4	0.40 ± 0.04	+48.2 ± 0.7	9.51 ± 0.03	300 ± 19	0.52 ± 0.02	+46.4 ± 1.6	9.62 ± 0.04	286 ± 13.8	0.46 ± 0.02	+46.7 ± 1.6	9.58 ± 0.06
	**ML2**	320 ± 10.5	0.50 ± 0.01	+45.2 ± 1.3	14.42 ± 0.04	330 ± 21	0.55 ± 0.03	+44.2 ± 1.4	14.43 ± 0.02	346 ± 16.2	0.56 ± 0.06	+43.8 ± 2.2	14.46 ± 0.05
	**ML3**	360 ± 16.8	0.54 ± 0.02	+41.1 ± 1.9	18.73 ± 0.02	385 ± 16.8	0.48 ± 0.04	+42.3 ± 0.6	18.71 ± 0.05	372 ± 6.7	0.68 ± 0.04	+38.4 ± 1.5	18.71 ± 0.04

## Data Availability

The data and supportive information are available within the article.

## LIST OF ABBREVIATIONS

AIC: Akaike Information Criterion
ATCC: American Type Culture Collection
BJ: Healthy Human Dermal Fibroblast Cell Lines.
CDCl3: Deuterated Chloroform
CYP19A1: Cyp19a1 Gene
DL%: Drug Loading
DMEM: Dulbecco's Modified Eagle Medium
DMSO: Dimethyl Sulfoxide
DSC: Differential Scanning Calorimetry
EE%: Encapsulation Efficiency
EPR: Enhanced Permeability And Retention
ERS100: Eudragit^®^ Rs 100
FT-IR: Fourier-transform Infrared Spectroscopy
HCC: Hepatocellular Carcinoma
1H-NMR: Proton Nuclear Magnetic Resonance
HepG2: Human Hepatocellular Carcinoma Cell Lines.
HPLC: High-performance Liquid Chromatography
K: Release Rate Constant kDa = Kilo Dalton
LTZ: Letrozole
MAPK/ERK: Mitogen-activated Protein Kinase/Extracellular Signal-regulated Kinase
ML1: Formulation Code
ML2: Formulation Code
ML3: Formulation Code
Ml-PL: Placebo Formulation Code
MSC: Model Selection Criterion
MTOR: Mechanistic Target of Rapamycin
MTT: Methylthiazolyl-Diphenyl-tetrazolium Bromide
PDI: Polydispersity Index
PNPS: Polymeric Nanoparticles
PTFE: Polytetrafluoroethylene r^2^ = Coefficient Of Determination
SD: Standard Deviation
SEM: Scanning Electron Microscopy
THLE-2: A Non-Tumorigenic Human Liver Epithelial Cell Culture Model
